# Autonomic Cardiovascular Responses in Acclimatized Lowlanders on Prolonged Stay at High Altitude: A Longitudinal Follow Up Study

**DOI:** 10.1371/journal.pone.0084274

**Published:** 2014-01-03

**Authors:** Priyanka Dhar, Vijay K. Sharma, Kalpana B. Hota, Saroj K. Das, Sunil K. Hota, Ravi B. Srivastava, Shashi B. Singh

**Affiliations:** 1 Defence Institute of High Altitude Research, Defence Research & Development Organisation, C/o 56 APO, Leh-Ladakh, Jammu and Kashmir, India; 2 Defence Institute of Physiology and Allied Sciences, Defence Research & Development Organisation, Lucknow Road, Timarpur, Delhi, India; University of Louisville, United States of America

## Abstract

Acute exposure to hypobaric hypoxia at high altitude is reported to cause sympathetic dominance that may contribute to the pathophysiology of high altitude illnesses. The effect of prolonged stay at high altitude on autonomic functions, however, remains to be explored. Thus, the present study aimed at investigating the effect of high altitude on autonomic neural control of cardiovascular responses by monitoring heart rate variability (HRV) during chronic hypobaric hypoxia. Baseline electrocardiography (ECG) data was acquired from the volunteers at mean sea level (MSL) (<250 m) in Rajasthan. Following induction of the study population to high altitude (4500–4800 m) in Ladakh region, ECG data was acquired from the volunteers after 6 months (ALL 6) and 18 months of induction (ALL 18). Out of 159 volunteers who underwent complete investigation during acquisition of baseline data, we have only included the data of 104 volunteers who constantly stayed at high altitude for 18 months to complete the final follow up after 18 months. HRV parameters, physiological indices and biochemical changes in serum were investigated. Our results show sympathetic hyperactivation along with compromise in parasympathetic activity in ALL 6 and ALL 18 when compared to baseline data. Reduction of sympathetic activity and increased parasympathetic response was however observed in ALL 18 when compared to ALL 6. Our findings suggest that autonomic response is regulated by two distinct mechanisms in the ALL 6 and ALL 18. While the autonomic alterations in the ALL 6 group could be attributed to increased sympathetic activity resulting from increased plasma catecholamine concentration, the sympathetic activity in ALL 18 group is associated with increased concentration of serum coronary risk factors and elevated homocysteine. These findings have important clinical implications in assessment of susceptibility to cardio-vascular risks in acclimatized lowlanders staying for prolonged duration at high altitude.

## Introduction

The autonomic nervous system (ANS) plays an important role in the regulation of a number of physiological processes during normal and pathophysiological conditions. Alteration in the ANS responses has been associated with the progression of cardiovascular diseases [1]. The ANS is influenced by both extrinsic factors like environment, stress etc as well as intrinsic factors that include hormonal changes [2]. Alteration in autonomic response is generally manifested through alterations in heart rate variability (HRV) [3], [4]. Several investigators have used HRV analysis to assess autonomic functions in field study set ups owing to its advantages like portability, non-invasiveness, faster data acquisition, cost-effectiveness and reliability [5]–[8]. Environmental stress like acute exposure to hypoxia at high altitude is reported to diminish linear HRV and increased nonlinear HRV thereby resulting in alterations in autonomic functions of the nervous system [9]. Though there is reduction in both high and low frequency band power at high altitude, the low to high frequency power ratio increases [10]–[12].

The effect of rapid ascent to high altitude on autonomic cardiovascular modulation and its relationship with spectral components of HRV during AMS has been previously documented [13], [14]. Studies by Bernardi et al. (2007) have shown exaggerated sympathetic activation in subjects with AMS while sympathetic activation was reduced in Himalayan high altitude natives [15]. Even after long-term acclimatization at sea level, high-altitude natives showed lower sympathetic activation, indicating a persisting high altitude adaptation [16]. Comparative evaluation of the influence of autonomic nervous system on heart and peripheral circulation in native high-altitude residents and sea-level residents at high altitude showed that even after acclimatization for one week, lowlanders showed sympathetic activation and skin vasoconstriction, while native highlanders residents did not show reduced vagal tone when compared to sea-level residents [17]. Intermittent exposure to hypoxia in athletes unacclimatized to high altitude on the other hand resulted in increased LF/HF ratio which is a determinant of sympatho-vagal balance and signifies sympathetic over-activity [18]. Previous studies have also demonstrated sympathetic dominance in resting subjects staying for a period of one month at extreme altitude of 5050 m [19]. Power spectral analysis showed reduced HRV with a virtual increase in the low frequency (LF) component during exposure to high altitude [16], [20], signifying an increased sympathetic modulation of the sinus node in response to hypobaric hypoxia. Over activation of the sympathetic neural system has been reported in non-natives following exposure to hypobaric hypoxia at high altitude [21]. In addition, there are other studies that have shown persistent increase in sympathetic nerve activity and chemosensitivity even after short duration of exposure to normobaric hypoxia [22], [23]. A recent study by Prabhakaran and Tripathi (2011) showed autonomic modulation on acute exposure to hyperoxic hypobaria in simulated altitude of 4574 m [24]. However, most of these studies were performed to evaluate the consequence of acute and intermittent hypoxia on the autonomic nervous system and the effect of prolonged stay at high altitude on cardiovascular autonomic system remains ambiguous.

According to the report of World Health Organization (WHO), approximately 35 million people travel to altitudes above 3000 m each year [25]. A large number of lowlander population stay for long durations at high altitude due to occupational requirements and call of duty. Despite considerable research on the adverse effects of high altitude on human health, information on effect of prolonged stay at high altitude on the physiological functions of lowlanders is relatively sparse. The present study therefore aimed at investigating the differential temporal regulation of autonomic responses in acclimatized lowlanders (sea level populations staying at high altitude after acclimalization) on prolonged stay at high altitude. The alterations in autonomic response in acclimatized lowlanders on different durations of stay at high altitude were evaluated through longitudinal follow up on a relatively large cohort. With an objective to determine the metabolic correlates for the sympatho-vagal response at high altitude, we estimated serum concentration of coronary risk factors including cholesterol, triglycerides, high density lipoprotein (HDL), low density lipoprotein (LDL) and very low density lipoprotein (VLDL). The possible influence of secondary hypertension due to kidney malfunction that could influence the autonomic response was also investigated by estimating creatinine and blood urea nitrogen [26]. Liver function tests comprising of alanine aminotransferease (ALT) and aspartate aminotransferase (AST) as well as serum folic acid, vitamin B_12_ and homocysteine levels were also estimated [27]–[30]. Concentration of serum angiotensin converting enzyme (ACE) and angiotensin II (Ang II) and plasma catecholamines *viz*. norepinephrine and epinephrine was also determined [31], [32].

## Materials and Methods

### Ethical clearance

The experimental protocol was approved by the ethics committee on human investigation of Defence Institute of High Altitude Research (DIHAR), Defence Research and Development Organisation (DRDO), Leh-Ladakh, India in accordance to Indian Council of Medical Research (ICMR) guidelines, and informed written consent was obtained from all the volunteers prior to enrollment.

### Study protocol and volunteers

The study was conducted in actual field conditions in Ladakh region, during August 2009-March 2012. Volunteers who had stayed for more than 24 months at sea level were enrolled at Lalgarh Jattan, Rajasthan (<250 m MSL) in May-July 2010. The volunteers were explained about the study purpose, protocol and expected outcomes and informed consent was obtained. Preliminary screening was performed based on eligibility criterion L1 comprising of age, gender, education, monthly income and physical and physiological ailments. A medical questionnaire comprising questions related to occurrence of chronic diseases, physical and physiological ailments, heart problems, stroke, epilepsy, head injury, drug abuse, psychological disorders and general health status was administered to all the volunteers. Volunteers were then screened for compliance to eligibility criteria L2 comprising of core behavioral measures (CBM) like core alcohol consumption (section A), core tobacco use (section C), core diet (section D) and core physical activity (section P) in accordance with WHO guidelines [33] and with Beck Depression Inventory (BDI) score [34] to investigate the presence of hitherto undetected depression (Table 1). The information was verified from a close acquaintance of the volunteer. Lake Louis Score for acute mountain sickness (AMS) was administered to the participants at high altitude to negate possible occurrence of AMS symptoms.

**Table 1:** Baseline characteristics of volunteers included in the study.

Level 1 (L1) Criteria	
Parameter	Criteria
**1 Age and anthropometric measures**	
Age (years)	23–35
Gender	Male
Education (years)	12±2*
Monthly earnings (INR)	18000±2500*
**2 Medical history/Health status**	
Any serious health illnesses	NA
Head injury resulting in loss of consciousness	NA
Any form of seizures, delirium tremens or convulsions	NA
Heart attack or any heart problem	NA
Cancer	NA
Allergies to medications, foods, animals, chemicals, or other agent	NA
Lung diseases such as asthma, emphysema, or chronic bronchitis	NA
Surgeries or hospitalizations	NA
Hypertension	NA
Diabetes	NA
Viral Hepatitis	NA
Dementia/Memory Impairment	NA
Stroke/Infarction/Cerebral Hemorrhage	NA
Kidney Disease	NA
GERD symptoms	NA
Chest Pain	NA
Congenital Heart Disease	NA
Neurological Problem/Epilepsy	NA
Familial Disorders	NA
**Level 2 (L2) Criteria (Core Behavioral Measures)**	
**Parameter**	**Criteria**
**1 Alcoholism**	Non-alcoholics
**2 Smoking of tobacco products**	Non-smokers
**3 Diet**	Vegetarian and non-vegetarian
**4 Physical activity**	Mild to moderate
**BDI Score**	4.85±3.96*

*Plus-minus values are mean ± SD; NA indicates not applicable.

**Figure 1:**
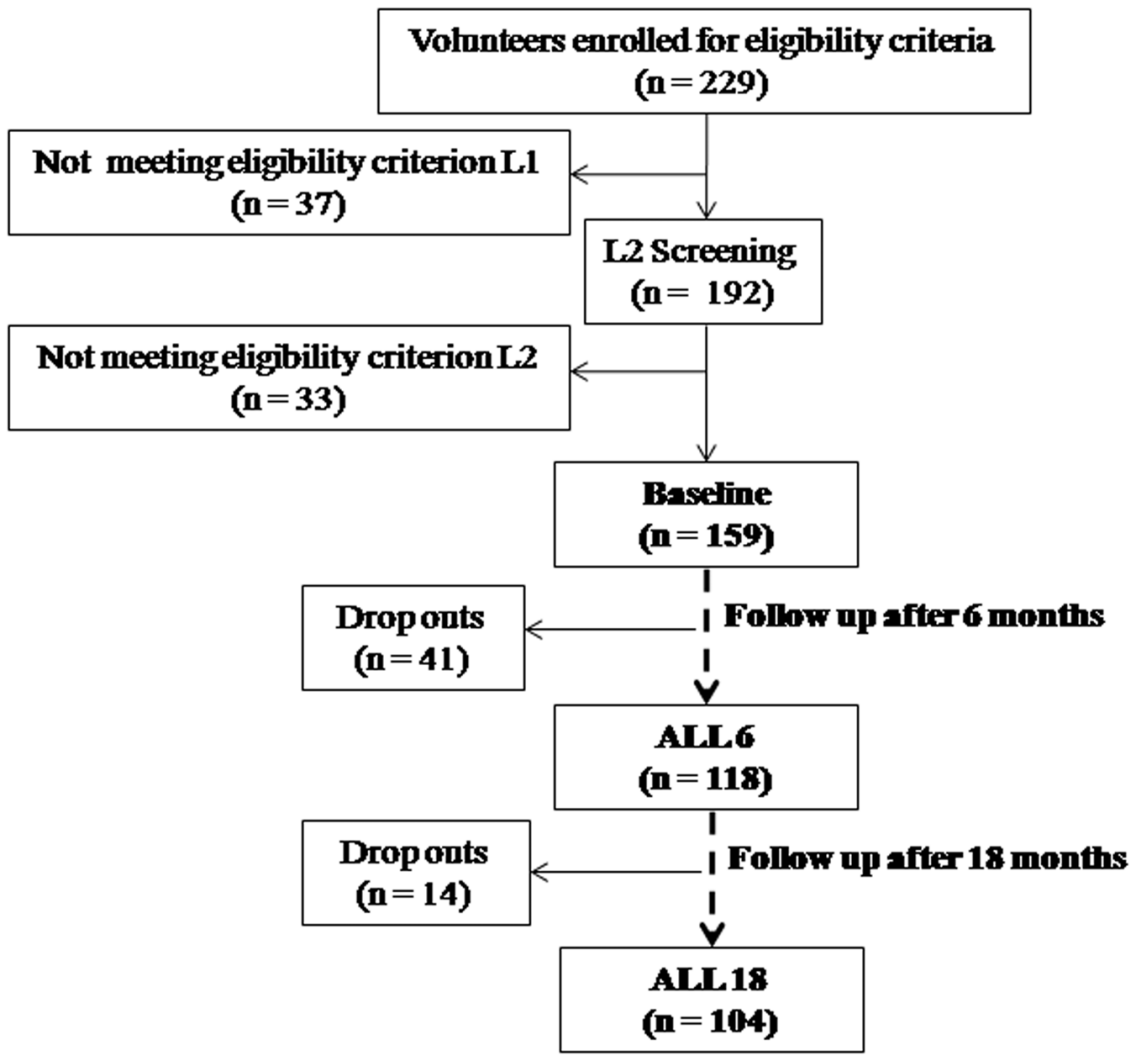
Study profile: Flow chart of the volunteers depicting recruitment, assessment and retention in the study.

Out of 229 volunteers between age group 23–35 of Indo-European origin who enrolled for the study, 159 volunteers qualified both L1 and L2 criterion and underwent complete investigation during acquisition of baseline data at MSL (<250 m) in Rajasthan region. Following induction of the study population to high altitude (4500–4800 m) in Ladakh region of India, only 118 volunteers could be followed up after 6 months (ALL 6) and finally 104 volunteers who constantly stayed at high altitude for 18 months qualified for the final follow up after 18 months (ALL 18), the remaining being drop outs or de-inductees (Fig. 1). In both phases, volunteers at high altitude were considered to be acclimatized when Lake Louise scores were <2 [35]. The physical activity was maintained at a constant level for all the volunteers throughout the duration of the study to negate the influence of physical activity of cardiac autonomic responses.

### Physiological measurements

Systolic and diastolic blood pressure (SBP and DBP), pulse rate (PR) and hemoglobin oxygen saturation (SpO_2_) were obtained in sitting position after 5 minutes rest prior to the investigation. Height was measured using a portable anthropometer. Body weight was measured using a portable digital scale (Omron Digital Weight Scale HN-286, Omron Healthcare Co., Kyoto, Japan). Body mass index (BMI) was calculated and expressed as kg/m^2^.

### ECG data acquisition and analysis

For recordings on ECG and respiration rate, volunteers were made to relax in sitting position for 15 minutes for habituation to the instrument and test environment. Recordings were acquired using BioHarness Physiology Monitoring System (Zephyr BioHarness data acquisition system with 3-lead configuration, BIOPAC Systems, Inc., 42 Aero Camio, Goleta, CA, USA) with MP 150 hardware and AcqKnowledge 3.9.1030 software. Raw data was acquired for 10 min with 250 Hz sample rate and post acquisition data processing was performed using AcqKnowledge 4.0.0 software. Artifacts, cumulative RR periods, and extra systoles were manually processed by calculation of interpolated or extrapolated values. The HRV indices with power spectral analyses were calculated by HRV analysis software 1.1 from the surface electrocardiogram [36]. The time-domain parameters *viz.* mean HR, mean RR, SDNN, RMSSD, NN50 and pNN50 were calculated directly from the raw RR interval time series. RR triangular index and TINN were calculated as geometric measures of HRV. SD1 was measured as the non-linear parameter of short-term HRV from Poincaré plot. In frequency-domain analysis power spectral density (PSD) of RR series was calculated by nonparametric Fast Fourier Transform (FFT) method. The parameters for frequency-domain included powers of LF, HF and TP in absolute values, normalized power of LF and HF bands, and the LF to HF ratio.

### Biochemical estimations

Serum and plasma were obtained from fasting blood samples collected from the volunteers in morning hours under aseptic conditions. Cholesterol, triglycerides, high density lipoprotein (HDL), low density lipoprotein (LDL), cholesterol/HDL, LDL/HDL and very low density lipoprotein (VLDL) were measured by CHOD-POD, GPO-POD and direct enzyme clearance methods [37]–[40] to assess coronary risk profile. Hemoglobin (Hb) was estimated by converting all forms of hemoglobin to colored cyanomethemoglobin and measured by a colorimeter while hematocrit (Hct) was measured using hematocrit reader according to previously described protocol [41]. Homocysteine, folic acid and vitamin B_12_ were measured by chemiluminesence method [42]–[44]. Creatinine and blood urea nitrogen (BUN) were measured spectrophotomentrically with Jaffe's kinetics and Urease method respectively [45], [46]. Alanine aminotransferase (ALT) and aspartate aminotransferase (AST) were measured spectrophotometrically [47]. Serum angiotensin converting enzyme (ACE) concentration was estimated by Boster's human ACE ELISA kit (Wuhan Boster Biological Technology Ltd., Wuhan, China). Serum angiotensin II concentration was estimated spectrophotometrically using RayBio® angiotensin II Enzyme Immunoassay (EIA) kit (Ray Biotech, Inc., Norcross, GA, USA). Plasma epinephrine and norepinephrine concentration were estimated by ELISA kit according to the instructions of the manufacturer (Abnova, Neihu District, Taipei City, Taiwan).

### Statistical analysis

Data acquired from only those individuals who participated throughout the study was considered for statistical analysis. Mean ± SEM was calculated for each group. Statistical analysis was performed using ANOVA with Duncun's Post Hoc test for comparisons between groups using SPSS 17.0 Statistics software (SPSS, Chicago, IL, USA). Relations between variables were analyzed by calculating the Pearson product-moment correlation coefficients. *P*-values<0.05 (two tailed) were considered to be significant. All the raw data was archived in the laboratory and a copy of the same was submitted to central records keeping centre of DIHAR.

## Results

### Physiological measures

SBP and DBP values were within the normal physiological range during the baseline and follow ups. Pulse rate was significantly higher in ALL 6 and ALL 18 when compared to the baseline values. The SpO_2_ at ALL 6 and ALL 18 significantly reduced when compared with the baseline data. ALL 6 showed increased pulse rate and reduced SpO_2_ when compared to ALL 18. BMI remained within the normal physiological range during baseline and follow ups (Table 2).

**Table 2:** Physiological measures of the study groups (n = 104).

	Normal range	Baseline	ALL 6	ALL 18
SBP (mm Hg)	110–130	120.86±0.58	123.18±0.72	123.05±0.80
DBP (mm Hg)	75–85	80.02±0.34	82.72±0.70	81.99±0.83
Pulse Rate (BPM)	70–80	71.76±0.51	85.17±1.12^*^	80.22±1.20^*#^
SpO_2_ (%)	95–98	96.44±0.09	89.18±0.20^*^	91.69±0.35^*#^
BMI	18.50–24.90	21.78±0.12	21.84±0.14	22.02±0.24

Values indicated are means ± SEM.

*P*<0.05: * compared with Baseline; # compared with ALL 6.

SBP: systolic blood pressure; DBP: diastolic blood pressure; SpO_2_: hemoglobin oxygen saturation; BMI: body mass index.

### Autonomic responses

ECG data analysis showed progressive shortening of overall HRV indicators *viz.* mean RR, SDNN, RRTI, TINN and TP in ALL 6 and ALL 18 when compared to the baseline study group. The indicators of sympathetic activity *viz.* mean HR and LF (nu) showed significantly higher values in ALL 6 and ALL 18 when compared with the baseline data. The parasympathetic activity predictors, such as RMSSD, NN50, pNN50, SD1, HF power and HF (nu) also significantly decreased in the ALL 6 and ALL 18 in comparison to the baseline values. The LF/HF ratio (sympatho-vagal balance) increased significantly in the ALL 6 and ALL 18 when compared with the baseline data. Respiration rate was also found to be increased significantly in the ALL 6 and ALL 18 in comparison with baseline. Sympathetic activity was maximum in ALL 6 along with lowest overall HRV and significant reduction in parasympathetic response when compared to baseline as well as ALL 18 (Table 3).

**Table 3:** Heart rate variability indices of the study groups (n = 104).

	Baseline	ALL 6	ALL 18
**Overall HRV measures**			
Mean RR (s)	0.792±0.010	0.671±0.010^*^	0.718±0.008^*#^
SDNN (s)	0.049±0.002	0.032±0.001^*^	0.042±0.001^*#^
			
RR TI	0.123±0.004	0.059±0.003^*^	0.087±0.001^*#^
TINN (ms)	351.63±15.92	202.58±8.14^*^	292.76±6^*#^
			
TP ms^2^	1566.58±94.60	748.42±43.62^*^	996.05±29.13^*#^
**Sympathetic activity measures**			
Mean HR (1/min)	77.44±0.88	90.24±0.98^*^	85.65±0.98^*#^
LF power ms^2^	671.68±34.28	412.18±23.11^*^	530.36±16.81^*#^
LF (nu)	59.06±0.98	80.12±0.76^*^	69.39±1.19^*#^
**Parasympathetic activity measures**			
RMSSD (ms)	39.01±2.74	21.10±0.89^*^	32.24±0.56^*#^
NN50 (count)	106.15±8.72	30.12±3.84^*^	71.61±2.98^*#^
pNN50 (%)	17.72±1.50	5.80±0.44^*^	11.15±0.90^*#^
SD1 (ms)	34.82±1.95	15.70±0.72^*^	24.99±0.57^*#^
HF power ms^2^	468.88±36.92	185.82±15.74^*^	289.12±8.73^*#^
HF (nu)	34.51±0.98	16.40±0.91^*^	24.59±0.66^*#^
**Sympatho-vagal balance measure**			
LF/HF	2.21±0.08	3.62±0.18^*^	3.07±0.11^*#^
**Respiration rate**	11.70±0.07	13.17±0.10^*^	13.22±0.11^*^

Values indicated are means ± SEM.

*P*<0.05: * compared with Baseline; # compared with ALL 6.

Mean RR: mean RR interval; SDNN: standard deviation of RR intervals; RMSSD: root mean square of the differences between consecutive RR intervals; NN50: number of consecutive RR intervals differing more than 50 ms; pNN50: percentage value of NN50 intervals; RRTI: RR triangular index; TINN: triangular index of normal to normal intervals; VLF: very low frequency; LF: low frequency; HF: high frequency.

### Biochemical and molecular changes in the serum

Hemoglobin and hematocrit increased significantly in ALL 6 and ALL 18 when compared with baseline values. ALL 18 showed highest hemoglobin and hematocrit concentration during the follow up study. BUN, creatinine, ALT and AST values remained within the normal reference range during the baseline and follow ups (Table 4).

**Table 4:** Hemoglobin, hematocrit, kidney and liver function profiling of the study groups (n = 104).

	Reference range	Baseline	ALL 6	ALL 18
Hb (g/dL)	13.80–17.20	15.28±0.07	17.78±0.06^*^	18.47±0.11^*#^
Hct (%)	43–52	46.88±0.17	53.56±0.22^*^	55.34±0.29^*#^
BUN(ng/ml)	6–20	9.22±0.24	9.38±0.23	9.42±0.25
Creatinine (ng/ml)	0.90–1.30	0.910±0.012	0.928±0.014	0.930±0.015
ALT (ng/ml)	30–65	43.12±0.81	44.19±1.02	43.46±1.39
AST (ng/ml)	15–37	29.22±0.28	29.50±0.40	29.90±1.25

Values indicated are means ± SEM.

*P*<0.05: * compared with Baseline; # compared with ALL 6.

Hb: Hemoglobin; Hct: Hematocrit; BUN: blood urea nitrogen; HDL: high density lipoprotein; LDL: low density lipoprotein; VLDL: very low density lipoprotein; ALT: alanine aminotransferase; AST: aspertate aminotransferase.

**Figure 2:**
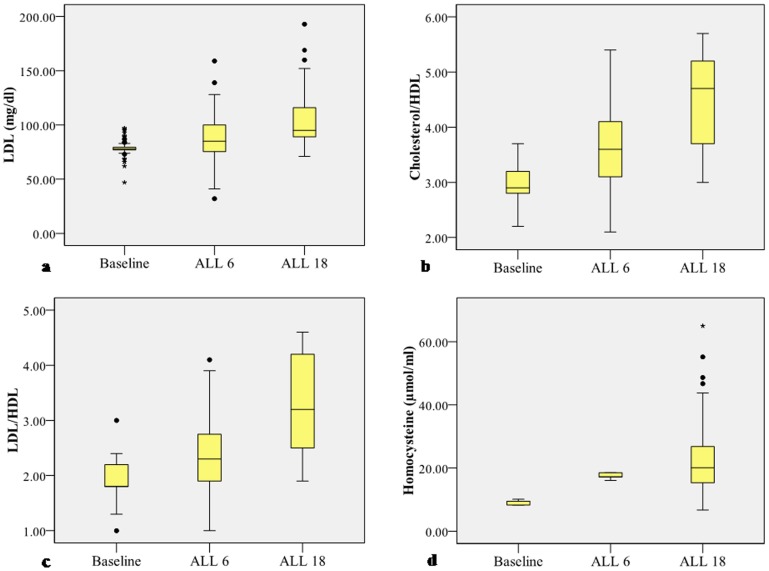
Graphic representation of serum coronary risk factor profile in baseline and follow up groups. The limits of the boxes represent the middle 50% of the data values; the extent of the lines encompass the interquartile range with extreme outlying data points shown as such. The central line within each box represents the median. **a.** Serum LDL concentration is significantly elevated in ALL 18 (*P*<0.05) vs Baseline. **b.** Serum cholesterol/HDL ratio is significantly elevated in ALL 18 (*P*<0.05) vs Baseline. **c.** Serum LDL/HDL ratio is significantly elevated in ALL 18 (*P*<0.05) vs Baseline. **d.** Serum homocysteine concentration is significantly elevated in ALL 18 (*P*<0.05) vs Baseline.

Results of serum coronary risk factor profiling have been described in Table 5. Serum cholesterol, triglycerides, HDL, VLDL, vitamin B_12_ and folic acid concentration were within the normal reference range. However, serum LDL, cholesterol/HDL and LDL/HDL concentration increased significantly beyond the reference range in ALL 18 and was higher in comparison to baseline and ALL 6 values. Serum homocysteine concentration was also significantly higher than the reference range in ALL 6 and ALL 18 (Table 5, Fig. 2). Serum homocysteine concentration was significantly higher in the ALL 18 population in comparison to ALL 6.

**Table 5:** Serum coronary risk factor profiling of the study groups (n = 104).

	Reference range	Baseline	ALL 6	ALL 18
Cholesterol (mg/dl)	<200	124.38±0.88	139.84±2.30	151.52±4.16
Triglycerides (mg/dl)	<150	81.22±1.80	98.54±2.85	129.46±7.36
HDL (mg/dl)	40–60	45.87±0.64	39.07±0.88	35.30±0.44
LDL (mg/dl)	<100	78.73±0.85	89.30±2.16	104.45±2.40^*#^
Cholesterol/HDL	3.30–4.40	2.98±0.03	3.64±0.07	4.47±0.08^*#^
LDL/HDL	0.50–3.0	1.92±0.03	2.37±0.07	3.27±0.08^*#^
VLDL (mg/dl)	≤30	15.98±0.42	19.12±0.86	26.50±1.17
Homocysteine (µmol/ml)	3.70–13.90	9.12±0.07	17.48±0.09^*^	22.97±1.18^*#^
Vitamin B12 (pg/mI)	211–911	262.82±3,78	224.81±3.86	213.70±5.37
Folic Acid (ng/ml)	>5.38	7.92±0.14	6.22±0.15	5.77±0.19

Values indicated are means ± SEM.

*P*<0.05: * compared with Baseline; # compared with ALL 6.

Hb: hemoglobin; Hct: hematocrit; BUN: blood urea nitrogen; HDL: high density lipoprotein; LDL: low density lipoprotein; VLDL: very low density lipoprotein; ALT: alanine aminotransferase; AST: aspertate aminotransferase.

**Figure 3:**
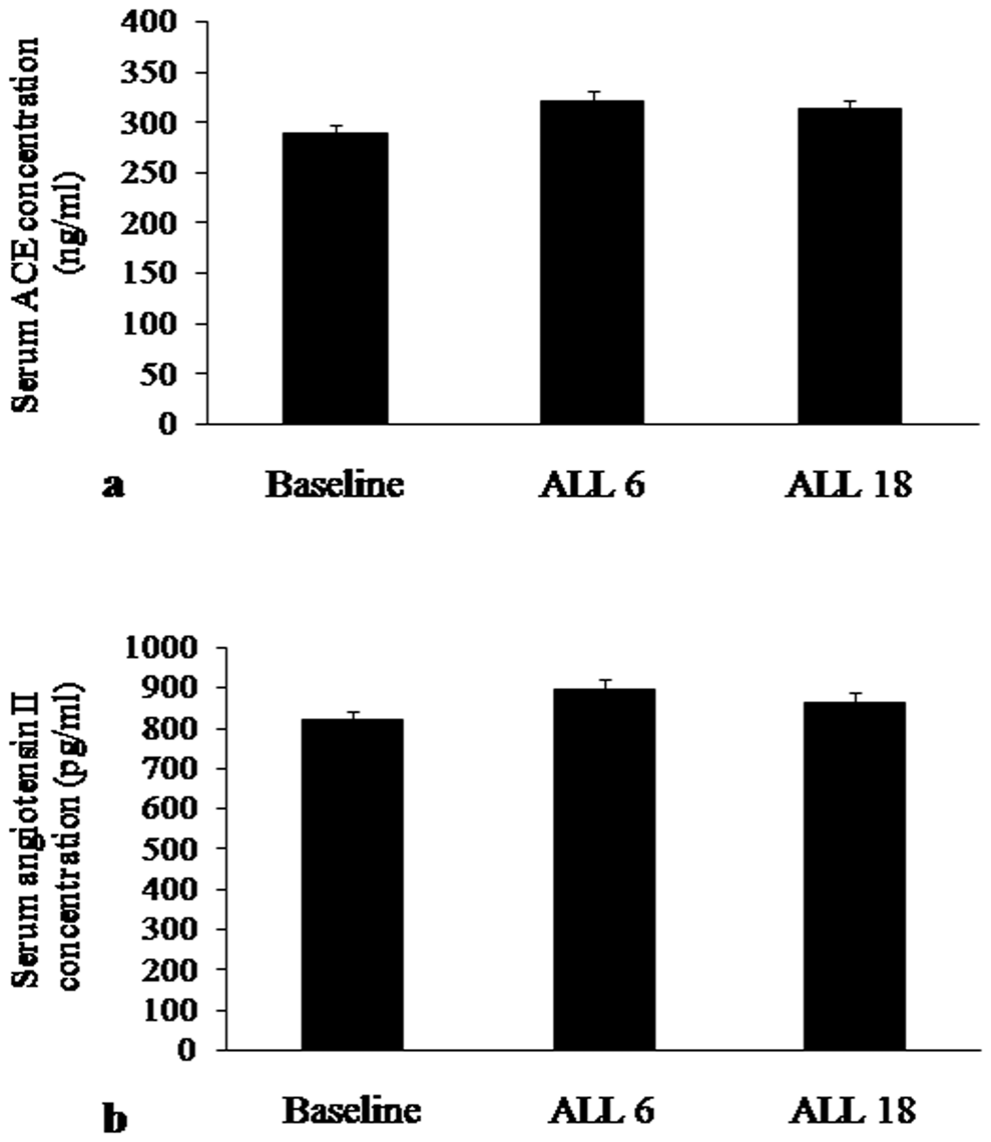
Concentration of a. Serum ACE (ng/ml) and b. Serum angiotensin II (pg/ml) in baseline and follow ups.

**Figure 4:**
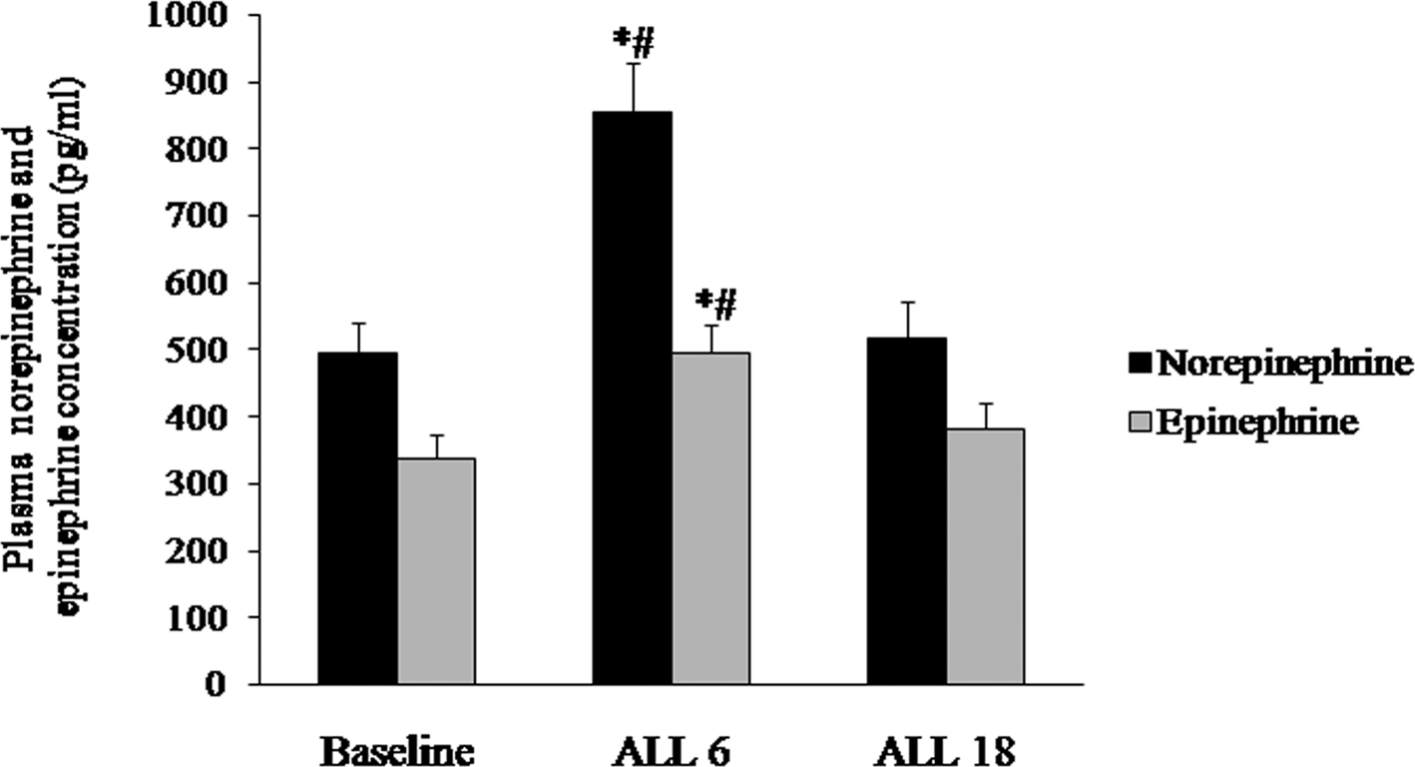
Plasma norepinephrine and epinephrine concentration (pg/ml) in baseline and follow ups. Plasma norepinephrine and epinephrine concentration are significantly elevated in ALL 6 vs Baseline and ALL 18. *P*<0.05: * compared with Baseline; # compared with ALL 18.

No significant change was observed in serum ACE and angiotensin II concentration during the follow ups (Fig. 3). Plasma norepinephrine and epinephrine concentration increased significantly in the ALL 6 population in comparison with baseline and ALL 18 population (Fig. 4).

## Discussion

The reduced partial pressure of oxygen on ascent to high altitude leads to decreased tissue oxygenation, an inimitable condition called hypobaric hypoxia, which culminates in a number of pathophysiological complications [48]–[52]. The function of ANS also gets adversely affected in hypobaric hypoxia at high altitude [12], [16], [17], [53]–[56]. Though acute high altitude exposure is reported to cause alterations in the cardiovascular system [57]–[59], the physiological response to prolonged hypobaric hypoxia has been less studied. In the present longitudinal study, increased pulse rate and reduced SpO_2_ was observed in the ALL 6 and ALL 18 groups in comparison with the baseline. Previous studies to determine the relationship between SpO_2_ and arterial blood pressure in healthy humans have attributed the decrease in SpO_2_ to increased blood pressure in persons with oxygen desaturation at high altitudes [60], [61]. Studies by Naeiji et al. (2010) have also shown increased heart rate even after acclimatization at high altitude [62]. Exposure to hypobaric hypoxia at high altitude leads to hypoxic pulmonary vasoconstriction that is responsible for the rise in pulmonary artery pressure [63]. Besides that, ascent to high altitude is also associated with reduction of pulmonary ventilatory function [64]. Our findings on increased pulse rate along with decreased SpO_2_ on prolonged stay at high altitude find support from previous reports on the rise in arterial blood pressure, increased pulse rate and lowering of SpO_2_ in subjects after acclimatization to hypobaric hypoxia at high altitude [65]–[73].

In recent years, HRV is being widely used for the assessment of ANS responses under diverse physiological and pathological conditions and has an array of clinical applications [74]. The heart rate variability method is amicable to field-type studies and has certain advantages *viz.* simplicity, portability, non-invasiveness, cost-effectiveness and reliability. Earlier studies clearly depict that measurement of HRV is a potent indicator of autonomic modulations at high altitude [9], [13], [14], [16], [19], [20]. In this current investigation, reduced HRV was observed during both the follow ups (ALL 6 and ALL 18) when compared to the baseline. Mean RR, SDNN, RRTI, TINN and TP that display the overall HRV were reduced significantly in the lowlanders at high altitude. RMSSD, NN50, pNN50, SD1, HF (ms^2^) and HF (nu) that account for the parasympathetic response of an individual, were also reduced significantly in the follow up groups at high altitude. The sympathetic activity measures *viz.* mean HR, LF (nu), and LF/HF ratio which signify the sympatho-vagal balance at the sinus node, increased in acclimatized lowlanders at high altitude in comparison with the baseline. These findings are an extension of previous reports on sympathetic dominance and reduction of parasympathetic activity following stay at high altitude for few days to few weeks [12], [14]–[16], [55], [75]–[83]. Studies by Bernardi et al. (1998), have shown decreased RR intervals and increased systolic blood pressure in sea level natives even after 7 days of stay at an altitude of 4970 m [16]. Similar studies conducted by Kanai et al. 2001, on untrained sojourners also showed a decrease in LF and HF even at altitudes of 3700 m [12]. Malhotra et al. (1976), during their investigations on effect of stay at high altitude for more than one year on the autonomic responses noted preponderance of the sympathetic activity even at an altitude of 3500 m [84]. The effect of such longer duration of stay at extreme altitudes above 4500 m, however, remained to be investigated. A unique study by Farinelli et al. (1994), on the effect of postural change on the heart rate, LF and HF of acclimatized lowlanders staying at extreme altitudes of 5050 m for one month showed decreased maximal heart rate and reduced sensitivity of the heart to adrenergic drive [75]. However, the limited number of 5 human subjects warrants a similar study in larger populations. Determination of autonomic activity by measuring muscle nerve sympathetic activity (MNSA) on non natives during exposure to hypobaric hypoxia showed overactivity of the sympathetic neural system [21]. Persistent increase in sympathetic nerve activity and chemosensitivity has also been reported, even after short duration exposure to normobaric hypoxia [22], [23].

We here report that, prolonged stay at extreme altitudes (>4500 m above sea level) for 6 months and 18 months results in a persistent sympathetic dominance when compared to the sea level populations. The large population size of the study provides strength to our findings on the altered autonomic response on shorter (6 months) and prolonged (18 months) stay at high altitude. Since we observed decrease in SpO_2_ of acclimatized lowlanders which showed no signs of improvement despite prolonged stay of 18 months, the increased sympathetic tone in the acclimatized lowlanders could therefore be a compensatory mechanism to ensure increased blood circulation to the peripherals under conditions of low oxygen saturation.

In addition to the autonomic changes, the hemoglobin and hematocrit concentration in the follow up groups were also found to increase significantly in comparison with the baseline. The increase in hemoglobin could be an adaptive physiological response to partially restore the arterial oxygen content which is crucial for altitude adaptation and results due to stimulated erythropoiesis [85]–[90]. The reduced heart rate in the ALL 18 population on prolonged duration of stay in high altitude that was observed during the present study could be due to an inverse relationship of hemoglobin and hematocrit with heart rate.

In the present study we observed an increase in serum LDL, cholesterol/HDL and LDL/HDL concentrations in ALL 18 along with significant reduction in HDL concentration. This is in contradiction to previous reports on lipid profile of healthy human subjects at moderate altitudes (1000–3500 m) which revealed that there was no risk of developing cardiovascular diseases due to dyslipidemia, reduced plasma total cholesterol, reduced LDL-c and increased level of HDL-c at moderate altitude [91]–[94]. Investigations on the effect of high altitude exposure on plasma lipids in a group of mountaineers showed reduction in LDL-c that was interpreted as an adaptive response to acute hypoxic exposure [95]. The discrepancy in the findings could be due to a higher altitude in which the study was conducted and long duration of stay at high altitude during the present study. Our findings find support from reports on increase of serum cholesterol, triglycerides and reduction of HDL-c at high altitude by Smith et al. (2011) and other researchers [96]–[98].

Besides increased susceptibility to hyperlipidemia induced cardiovascular diseases our findings also show significant increase in serum homocysteine concentration in the ALL 6 and ALL 18 when compared to baseline. Homocysteine concentration in the serum is influenced by genetic, nutritional, physiological and environmental factors [99]–[101]. Binding of nitric oxide (NO) with vitamin B_12_ and its precursors resulting in inhibition of methionine synthase activity could be a plausible reason for the increase in homocysteine that was observed in ALL>6 and ALL>18 groups during the present study [102], [103]. Our findings find support from previous reports on upregulation of nitric oxide synthase (NOS) and increase in NO following high altitude exposure [104], [105]. Our findings are of clinical relevance considering the fact that hyperhomocysteinemia is accepted to be an independent predictor of cardiovascular diseases [106]. Similar studies by Ashraf et al. (2006) have also revealed that high altitude stay could result into hyperhomocysteinemia as a risk factor for arterial and venous thrombosis. However, we observed that hyperhomocysteinemia at high altitude was independent of vitamin B_12_ and folic acid [107]. Previous studies by Tayama et al. (2006) have associated hyperhomocysteinemia to systemic arterial stiffness and greater blood pressure response to stress in hypertensive patients [108]. Increased oxidative stress has been previously demonstrated to play a pathophysiological role in the deleterious endothelial effects of homocysteine by promoting vasoconstriction and impairing acetylcholine mediated endothelium-dependent vasodilatation in resistance vessels [109].

The heart rate is controlled by the balance between sympathetic and parasympathetic nervous system activity, and the reduced parasympathetic activity is thought to be responsible for the elevation in heart rate during acute hypoxia [110]. With several investigators reporting increase in parasympathetic activity [111] while others reporting reduction in parasympathetic response [112] and certain reports showing that parasympathetic activity remains unaffected [113], the role of the parasympathetic activity in acute hypoxia is still a subject of debate. Koller et al. (1988) showed that both increased parasympathetic withdrawal and sympathetic stimulation is responsible for the resting heart rate elevation in healthy subjects exposed to a simulated altitude of 6000 m during acute hypoxic exposure [114]. The plasma catecholamine in particular has been reported to regulate the autonomic function. Prominent change in catecholamine concentration has been observed by several researchers following stay at high altitude. Increase in concentration of norepinephrine and epinephrine has been shown after 3–9 weeks of chronic high altitude exposure [115]–[117]. We also observed increased concentration of plasma norepinephrine and epinephrine in the ALL 6 population in comparison with the baseline and ALL 18. The increased sympathetic activity in association with reduced HRV in ALL 6 population could therefore be attributed to the increased plasma catecholamine concentration. However, after prolonged high altitude exposure of 18 months, the concentration of plasma catecholamine reduced to baseline level. On the contrary our results showed increased concentration of serum homocysteine and other coronary risk factors in ALL 18 when compared with baseline and ALL 6. Hyperhomocysteinemia is known to be an independent risk factor for systemic arterial stiffness and high blood pressure during stress [108]. Homocysteine could promote vasoconstriction and cause impairment of endothelium-dependent vasodilatation in blood vessels [109]. In the presence of normal catecholamine concentration, the reduced HRV after prolonged high altitude exposure for 18 months could be attributed to the increased concentration of serum coronary risk factors and elevated homocysteine. This hypothesis finds support from findings by Acampa et al., 2011 showing that hyperhomocytenemia is associated with an alteration in the electrical atrial conduction, possibly contributing, at least in part, to the increased risk of cardiac arrhythmias in the denervated hearts of orthotopic heart transplantation (OHT) patients [118]. However, in light of previous findings on negligible role of homocysteine in influencing sympathetic activity [119], further research is required to understand the factors that mediate sympathetic dominance on prolonged stay for >18 months at high altitude.

## Conclusion

Based on the findings of the present study, it may be interpreted that the autonomic response is greatly affected following high altitude exposure both for shorter (6 months) as well as prolonged (18 months) durations. However, the autonomic response appears to be regulated by two distinct mechanisms in the ALL 6 and ALL 18. While the autonomic alteration in the ALL 6 group is an outcome of increased sympathetic activity resulting from the increased plasma catecholamine concentration, the alteration of autonomic response in ALL 18 group could be associated with increased concentration of serum coronary risk factors and elevated homocysteine. These findings may also have important clinical implications in assessment of susceptibility to cardio-vascular risks in acclimatized lowlanders staying for longer durations at high altitude.
